# Supplementary material for: Anabolic Sensitivity in Healthy, Lean, Older Men Is Associated With Higher Expression of Amino Acid Sensors and mTORC1 Activators Compared to Young
Source: J Cachexia Sarcopenia Muscle. 2024 Nov 19;16(1):e13613. doi: 10.1002/jcsm.13613 (PMC11670179; doi:10.1002/jcsm.13613)
Supplement: Supplementary file 1 — Data S1 Supporting information [file JCSM-16-e13613-s003.docx]

**Supplemental references**

S1. [1]

S2. [2]

S3. [3]

S4. [4]

S5. [5]

1. Horwath O, Moberg M, Hirschberg AL, Ekblom B, Apró W. Molecular Regulators of Muscle Mass and Mitochondrial Remodeling Are Not Influenced by Testosterone Administration in Young Women. Front Endocrinol (Lausanne). 2022;13:874748.

2. Mascher H, Ekblom B, Rooyackers O, Blomstrand E. Enhanced rates of muscle protein synthesis and elevated mTOR signalling following endurance exercise in human subjects. Acta Physiol (Oxf). 2011;202:175-84.

3. Apro W, Moberg M, Hamilton DL, Ekblom B, Rooyackers O, Holmberg HC, Blomstrand E. Leucine does not affect mechanistic target of rapamycin complex 1 assembly but is required for maximal ribosomal protein s6 kinase 1 activity in human skeletal muscle following resistance exercise. FASEB J. 2015;29:4358-73.

4. Borno A, van Hall G. Quantitative amino acid profiling and stable isotopically labeled amino acid tracer enrichment used for in vivo human systemic and tissue kinetics measurements. J Chromatogr B Analyt Technol Biomed Life Sci. 2014;951-952:69-77.

5. von Haehling S, Coats AJS, Anker SD. Ethical guidelines for publishing in the Journal of Cachexia, Sarcopenia and Muscle: update 2021. J Cachexia Sarcopenia Muscle. 2021;12:2259-61.
