# Supplementary figures and images for: Anabolic Sensitivity in Healthy, Lean, Older Men Is Associated With Higher Expression of Amino Acid Sensors and mTORC1 Activators Compared to Young
Source: J Cachexia Sarcopenia Muscle. 2024 Nov 19;16(1):e13613. doi: 10.1002/jcsm.13613 (PMC11670179; doi:10.1002/jcsm.13613)

# Supplemental figure 1

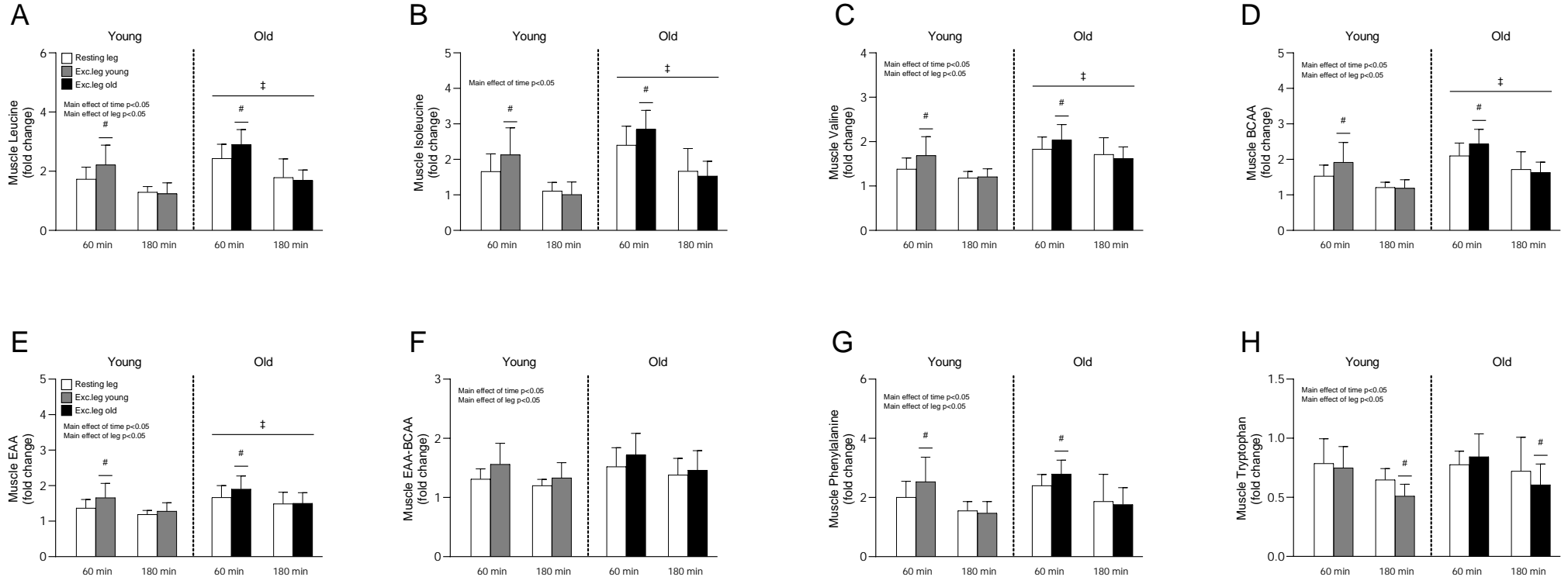

Supplement: Supplementary file 2 — Figure S1 Muscle concentrations of leucine (A), isoleucine (B), valine (C), BCAA (D), EAA (E), EAA‐BCAA (F), phenylalanine (G) and tryptophan (H) presented as fold changes before and after resistance exercise and intake of EAA in Young and Old. White bars represent the resting leg in both groups, whereas grey and black bars represent the exercising leg in Young and Old, respectively. A three‐way (age x time x leg) repeated‐measures ANOVA was used to analyse the data. The ANOVA revealed a significant interaction effect (time x leg) for (A, B, C, D, E, G, H), main effects of age for (A, B, C, D, E) and main effects of leg for (A, B, C, D, E, G, H). The symbols marked with short lines represent a two‐way interaction and symbols marked with a long line represent a main effect; * p < 0.05 different from baseline, # p < 0.05 different from the resting leg, ‡ p < 0.05 different from Young. Values are presented as means ± SD for 20 participants. For statistical analyses, data for supplemental Figure 1A‐F were log‐transformed [file JCSM-16-e13613-s002.pdf]

# Supplemental figure 2

A

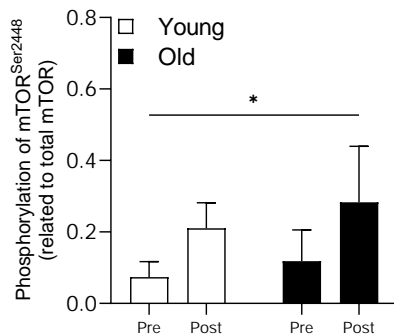

B

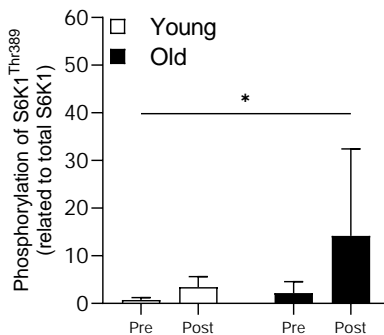

C

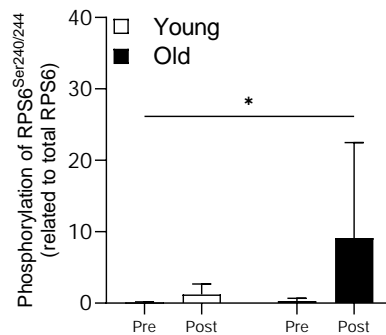

D

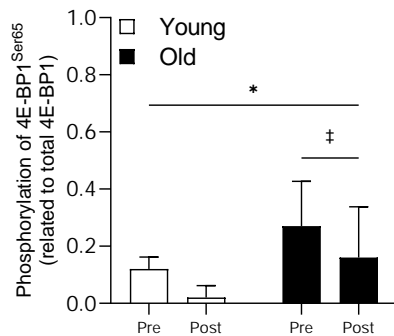

E

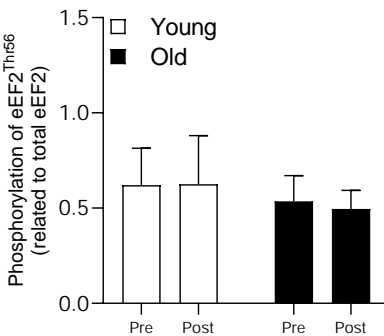

F

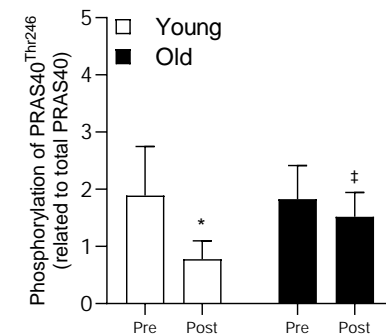

Supplement: Supplementary file 3 — Figure S2 Phosphorylation of mTOR at Ser2448 (A), S6K1 at Thr389 (B), RPS6 at Ser240/244 (C), 4E‐BP1 at Ser65 (D), eEF2 at Thr56 (E) and PRAS40 at Thr246 (F) before and immediately after resistance exercise in Young (white bars) and Old (black bars). A two‐way (age x time) repeated‐measures ANOVA was used to analyse the data. The ANOVA revealed a significant interaction effect (age x time) for (F), a main effect of time for (A, B, C, D) and a main effect of age for (D). The symbols marked without lines represent a two‐way interaction and symbols marked with lines represent a main effect. * p < 0.05 different from baseline or main effect of time, ‡ p < 0.05 different from Young. Values are presented as means ± SD for 20 participants. [file JCSM-16-e13613-s005.pdf]

## Cytosolic fraction

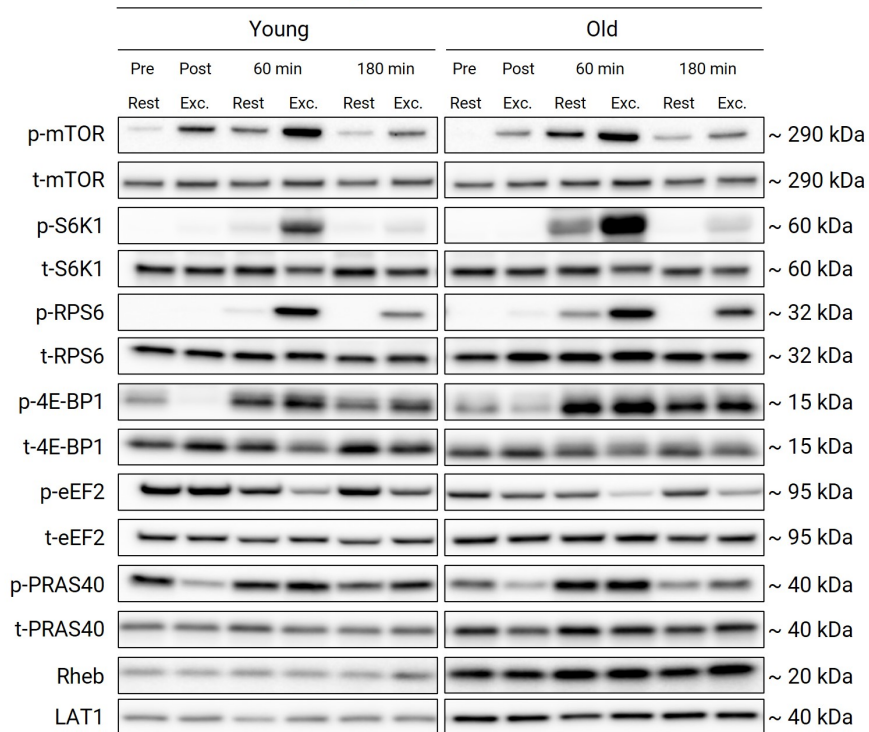

## Lysosomal fraction

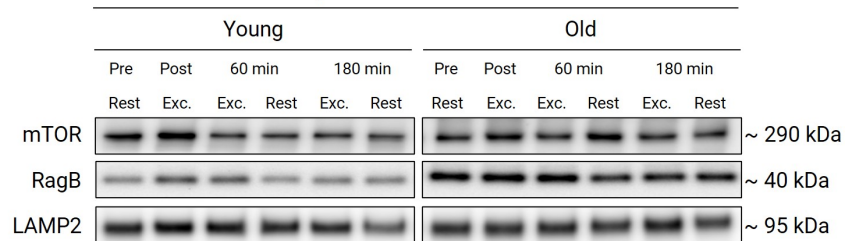

### Fraction purity

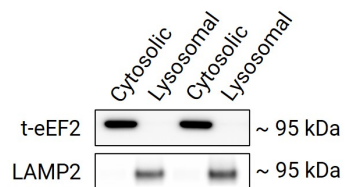

### Total protein stain (memcode)

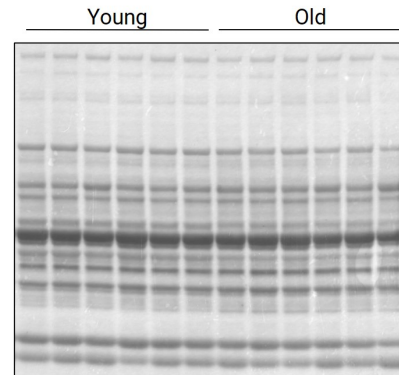

Supplement: Supplementary file 4 — Figure S3 Representative images of the immunoblots for the proteins presented in Figure 6 and 7. The left panel illustrates the phosphorylated and total forms of proteins residing in the cytosolic fraction and the right panel illustrates proteins residing in the lysosomal fraction. Representative blots are presented from one young and one old participant. In the bottom right panels, the purity of the two different fractions as well as the total protein stains (MemCode™ Reversible Protein Stain) are shown. [file JCSM-16-e13613-s004.pdf]

# Supplemental figure 4

## A

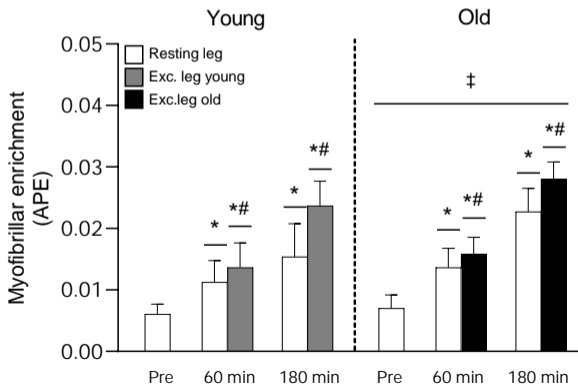

Supplement: Supplementary file 5 — Figure S4 Enrichment of L‐[ring 13C6] phenylalanine in the myofibrillar fraction in the resting leg and exercising leg of Young and Old (A). A three‐way (age x time x leg) repeated‐measures ANOVA was used to analyse the enrichment of the myofibrillar fraction. The ANOVA revealed a significant interaction effect (time x leg) and a main effect of age. The symbols marked with short lines represent a two‐way interaction and symbols marked with a long line represent a main effect; * p < 0.05 different from baseline, # p < 0.05 different from the resting leg, ‡ p < 0.05 different from Young. Values are presented as means ± SD for 20 participants. For statistical analyses, data for supplemental Figure 4 were log‐transformed. [file JCSM-16-e13613-s001.pdf]
